# Impact of dorzolamide, benzalkonium-preserved dorzolamide and benzalkonium-preserved brinzolamide on selected biomarkers of oxidative stress in the tear film

**DOI:** 10.1186/s12886-021-02079-y

**Published:** 2021-09-01

**Authors:** Lech Sedlak, Marta Świerczyńska, Weronika Borymska, Maria Zych, Dorota Wyględowska-Promieńska

**Affiliations:** 1 Department of Ophthalmology, Faculty of Medical Sciences in Katowice, Medical University of Silesia in Katowice, Katowice, Poland; 2 Department of Ophthalmology, Kornel Gibiński University Clinical Center, Medical University of Silesia in Katowice, Katowice, Poland; 3 Department of Pharmacognosy and Phytochemistry, Faculty of Pharmaceutical Sciences in Sosnowiec, Medical University of Silesia in Katowice, Sosnowiec, Poland

**Keywords:** Benzalkonium chloride, Dorzolamide, Brinzolamide, Carbonic anhydrase inhibitor, Oxidative stress, Ocular surface, Glaucoma

## Abstract

**Background:**

Long-term use of topical, especially benzalkonium chloride (BAC)-preserved, antiglaucoma medications can cause a negative impact on the ocular surface. The aim of the study was to assess the effect of topical carbonic anhydrase inhibitors (CAIs) on selected oxidative stress biomarkers in the tear film.

**Methods:**

The patients were divided into four sex-matched groups: group C (*n* = 25) – control group – subjects who did not use topical antiglaucoma medications, group DL (*n* = 14) – patients using preservative-free dorzolamide, group DL + BAC (*n* = 16) – patients using topical BAC-preserved dorzolamide, group BL + BAC (*n* = 17) – patients using BAC-preserved brinzolamide. Subjects in all the study groups have been using the eye drops two times daily for 6–12 months. The oxidative stress biomarkers in the tear film samples were measured: total protein (TP) concentration, advanced oxidation protein products (AOPP) content, total sulfhydryl (-SH) groups content, the activity of superoxide dismutase (SOD), catalase (CAT), and glutathione peroxidase (GPx), as well as Total Oxidant Status (TOS), Total Antioxidant Response (TAR), and Oxidative Stress Index (OSI).

**Results:**

The advanced oxidation protein products content, Total Oxidant Status as well as superoxide dismutase and catalase activities in the group DL + BAC and BL + BAC were higher in comparison with the group C. The total sulfhydryl groups content was lower in the group DL + BAC and BL + BAC when compared to group C. Oxidative Stress Index was higher in the groups DL + BAC and BL + BAC in comparison with the groups DL and C.

**Conclusions:**

Use of topical benzalkonium chloride-preserved carbonic anhydrase inhibitors increases oxidative stress in the tear film.

## Introduction

Glaucoma is a group of eye diseases representing a wide spectrum of clinical presentations and etiologies, which can lead to a permanent loss of visual function due to progressive degeneration of the retinal ganglion cells and nerve axons. The only established modifiable risk factor of glaucoma-associated optic neuropathy development is a reduction of elevated intraocular pressure (IOP), which can be obtained using topical eye drops or surgery [1]. Because of the chronic nature of the disease, antiglaucoma medications must be used over a long period. In turn, long-term exposure to the active ingredients and associated preservatives can lead to the development of ocular surface disease (OSD) or aggravate pre-existing abnormalities.

OSD is a constellation of disorders affecting the eyelids, conjunctiva and corneal surface, and the signs that appear in its course are largely resemblant to the side effects of antiglaucoma eye drops [2, 3]. Up to 59 % of patients treated for glaucoma or ocular hypertension report symptoms of OSD in at least one eye [3]. The severity of symptoms is correlated with the number of topical medications used to lower IOP as well as the presence of preservatives in the formulation [4].

The most common preservative, present in approximately 70 % of ocular medications, remains benzalkonium chloride (BAC), which is an effective antimicrobial agent with broad-spectrum activity. Additionally, its surfactant properties allow for the solubilization of ionic components of initially immiscible solvents and further stabilization of the substances [5–7]. On the other hand, it has been shown in vitro as well as in vivo, in both animals and humans, studies, that BAC-preserved eye drops have a deleterious impact on the ocular surface. Their application is associated with the tear film hyperosmolarity and instability, meibomian gland dysfunction, reduced density of goblet cells, corneal and conjunctival epithelial cell apoptosis, corneal nerves damage as well as inflammation of ocular tissues and fibrotic reaction [2–4, 7–19]. The incidence of ocular symptoms from a large pooled data set of glaucomatous patients (*n* = 9658) treated with topical preserved medications ranged between 27 and 52 % and was significantly higher than in the group of people using preservative-free formulations [20]. The clinical signs associated with the use of BAC-preserved eye drops include conjunctival hyperemia, superficial punctate keratitis, blepharitis, reduced tear production, increased osmolarity and abnormal tear film function. The above side effects can significantly affect the quality of life of patients, decrease compliance and, as a result, lead to disease progression [20, 21]. Therefore, selecting a substance that exerts a minimal effect on the ocular surface is crucial in glaucoma therapy.

The main classes of topical antiglaucoma medications include carbonic anhydrase inhibitors (CAIs): dorzolamide and brinzolamide. These are sulfonamides that lower IOP values by decreasing aqueous humor secretion via inhibition of carbonic anhydrase isoenzyme II in the ciliary processes. CAIs are most often used as adjuvant therapy to other antiglaucoma agents [22]. Assessing ocular comfort after installation of CAIs, dorzolamide compared to brinzolamide more often causes stinging, burning and redness due to non-physiological pH (5.6 vs. 7.5, respectively) and the use of sodium citrate as a buffer. On the other hand, brinzolamide contributes to a greater extent to blurred vision induced by the viscosity of the eye drop [23]. To our knowledge, a direct effect of BAC-preserved and unpreserved CAIs on oxidative stress in the tear film was not investigated so far.

Therefore, the aim of this study was to evaluate the influence of topical CAIs: preservative-free dorzolamide, BAC-preserved dorzolamide and BAC-preserved brinzolamide on total protein (TP) concentration, advanced oxidation protein products (AOPP) content, total sulfhydryl (-SH) groups content, the activity of superoxide dismutase (SOD), catalase (CAT), glutathione peroxidase (GPx), Total Oxidant Status (TOS), Total Antioxidant Response (TAR) and Oxidative Stress Index (OSI) in the tear film.

## Methods

### Study design and participants

The protocol of this cross-sectional study was approved by the Bioethical Committee of Medical University of Silesia (approval number: KNW/0022/KB1/87/17). All procedures were performed in accordance with the principles of the Declaration of Helsinki. Patients who met the inclusion were enrolled. Written informed consent was obtained from each patient.

The study was carried out among the patients of the Department of Ophthalmology and/or the Outpatient Clinic of the Kornel Gibiński University Clinical Centre, Medical University of Silesia in Katowice, Poland. The study group consisted of individuals older than 18 and younger than 70 years of age (mean age 54.89 ± 6.71) who were treated with one of three topical CAIs: (1) preservative-free dorzolamide, (2) BAC-preserved dorzolamide, (3) BAC-preserved brinzolamide.

The exclusion criteria comprised: (1) the use of any other eye drops, (2) the use of fixed-combination antiglaucoma medications, (3) the history of diabetes, autoimmune, allergic or thyroid disorders, (4) the history of refractive, intraocular or oculoplastic procedures, (5) the history of dry eye disease (Ocular Surface Disease Index (OSDI) score > 12), (6) the signs of anterior segment inflammation (conjunctivitis, keratitis, uveitis) or meibomian gland dysfunction, (7) the features of the ocular surface disease (the presence of the lid-parallel conjunctival folds (LIPCOF), corneal superficial punctate fluorescein staining, fluorescein break-up time (FBUT) > 10 s).

The control group included individuals who did not use any eye drops and did not meet the above exclusion criteria.

In total, 72 suitable patients (38 females and 34 males) were divided into 4 sex-matched groups:

- group C (*n* = 25; including 13 females and 12 males, mean age 52.88 ± 1.9) – control group – subjects who did not use topical antiglaucoma medications.
- group DL (*n* = 14; including 8 females and 6 males, mean age 54.92 ± 1.95) – patients with POAG treated with topical preservative-free dorzolamide (Nodofree, Polfa Warszawa).
- group DL + BAC (*n* = 16; including 8 females and 8 males, mean age 56.19 ± 1.7) – patients with POAG treated with topical BAC-preserved dorzolamide (Trusopt, Santen).
- group BL + BAC (*n* = 17; including 9 females and 8 males, mean age 55.82 ± 1.66) – patients with POAG treated with BAC-preserved brinzolamide (Azopt, Novartis).

Subjects in the groups DL, DL + BAC, BL + BAC have been using the eye drops two times daily for 6–12 months.

### Study Protocol for tear film collection

The tears were collected as described in [24]: 30 µl of saline was pipetted into the eye. Afterwards patients moved the closed eye for 5 s, then two glass capillary micropipettes (40 µl each) were placed at the lower tear meniscus to collect the tear film. The samples were then transferred into Eppendorf tubes and frozen immediately at -80 °C.

### Oxidative stress parameters estimation

The oxidative stress markers in the tear film samples were measured: TP concentration, AOPP content, -SH groups content, the activity of SOD, CAT and GPx, as well as TOS, TAR and OSI.

To evaluate AOPP, Witko-Sarsat’s protocol was employed. Samples of the tears were mixed with 1.16 M potassium iodide and then reactiuon was stopped by addition of glacial acetic acid. The samples were read at 340 nm and chloramine T was used as a standard curve [25]. Total -SH groups content was assayed based on Ellman’s protocol. In this method samples were mixed with phosphate buffer (pH = 8) and 5,5′-dithiobis-(2-nitrobenzoic acid, then after color development were measured at 412 nm. Calculations were made with extinction coefficient = 13,600 M/cm [26].

Two Erel’s protocols were used in order to evaluate TOS and TAR. In TOS method reagent 1 consisted of xylenol orange, NaCl and glycerol in sulphuric acid was added to the samples. This mixture was read at two wavelengths: 560 nm and 800 nm. After this measurement reagent 2 was added (solution of ferrous ions and o-dianisidine in sulphuric acid). After four minutes of incubation, the second read at the same wavelengths was taken. The results from measurement 1 were extracted from those from measurement 2 to obtain the differences which represented TOS in the tears. In this method hydrogen peroxide was used to prepare standard curve and the results are presented as H_2_O_2_: equivalent per 1 ml of the tears [27]. TAR was assayed as follows: to the tears samples reagent 1 was added (o-dianisidine and ferrous ions dissolved in Clark and Lubs solution) and this mixture was measured at 444 nm. Subsequently, reagent 2 (hydrogen peroxide in Clark and Lubs solution) was added and the mixture was left for 4 min for incubation, then read at 444 nm. The difference between measurement 2 and 1 represented TAR value presented as Trolox equivalent per 1 ml of tears [28].

TOS and TAR values were used to calculate oxidative stress index (OSI) according to equation: OSI = TOS/TAR.

TP level was assessed using the BioSystems colorimetric kit (BioSystems S.A. Costa Brava, Barcelona, Spain) according to the attached booklet. As far as the enzymatic assays are concerned, the commercially available kits were used (Cayman Chemical, Ann Arbor, Michigan, USA): Superoxide Dismutase (SOD) Assay Kit No. 706,002; Catalase (CAT) Assay Kit No. 707,002 and Glutathione Peroxidase (GPx) Assay Kit No. 703,102. All procedures were conducted according to the producer’s manuals.

### Statistics

All statistical analyses were performed using Statistica 10 Software (StatSoft, Tulsa, OK, USA). The obtained results were subjected to statistical analysis with one-way ANOVA. Since the data met the ANOVA assumptions of homogeneity of variance (Levene’s test) or normality (Shapiro-Wilk’s test), the Duncan’ post-hoc test was performed. Data are presented as arithmetic mean ± SEM and statistical significance was accepted for *p*-value < 0.05.

## Results

### Effect of topical CAIs on the total protein (TP) concentration in the tear film

**Fig. 1:**
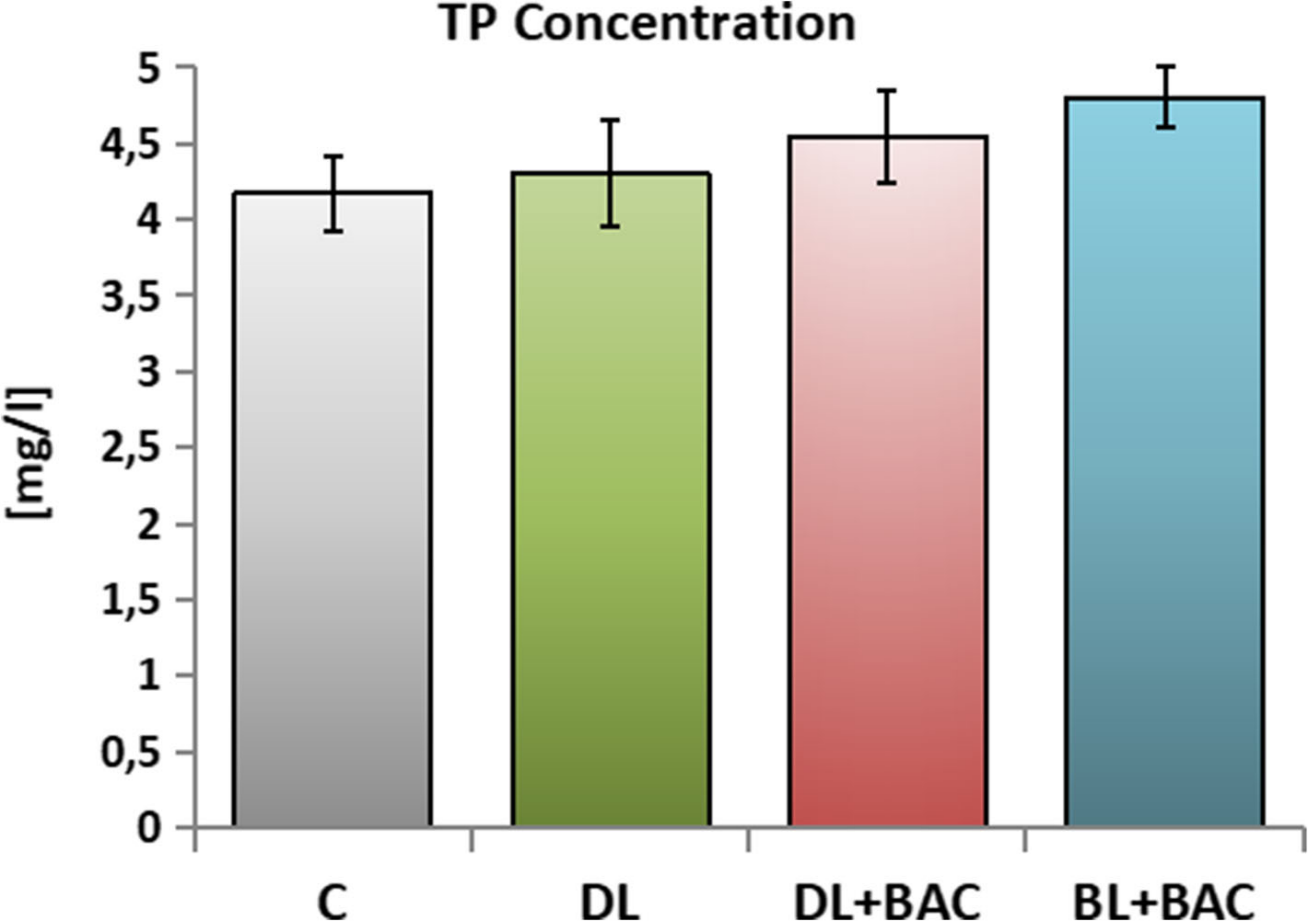
Effect of topical carbonic anhydrase inhibitors (CAIs) on the total protein (TP) concentration in the tear film. C – control group (*n* = 25); DL – patients with primary open-angle glaucoma (POAG) using topical dorzolamide (Nodofree, Polfa Warszawa) (*n* = 14); DL + BAC – patients with POAG using topical dorzolamide + benzalkonium chloride (BAC) (Trusopt, Santen) (*n* = 16); DL + BAC – patients with POAG using topical brinzolamide + BAC (Azopt, Novartis) (*n* = 17). Results are presented as means ± SEM

There were no differences in the TP concentrations in the tear film of patients using topical preservative-free dorzolamide (group DL) as compared to the group C. The TP concentration was found to be slightly higher in the group of patients using topical BAC-preserved medications (group DL + BAC and BL + BAC) when compared with group DL as well as with group C, but these differences were not statistically significant (Fig. 1).

### Effect of topical CAIs on the antioxidative enzymes activity in the tear film

**Fig. 2:**
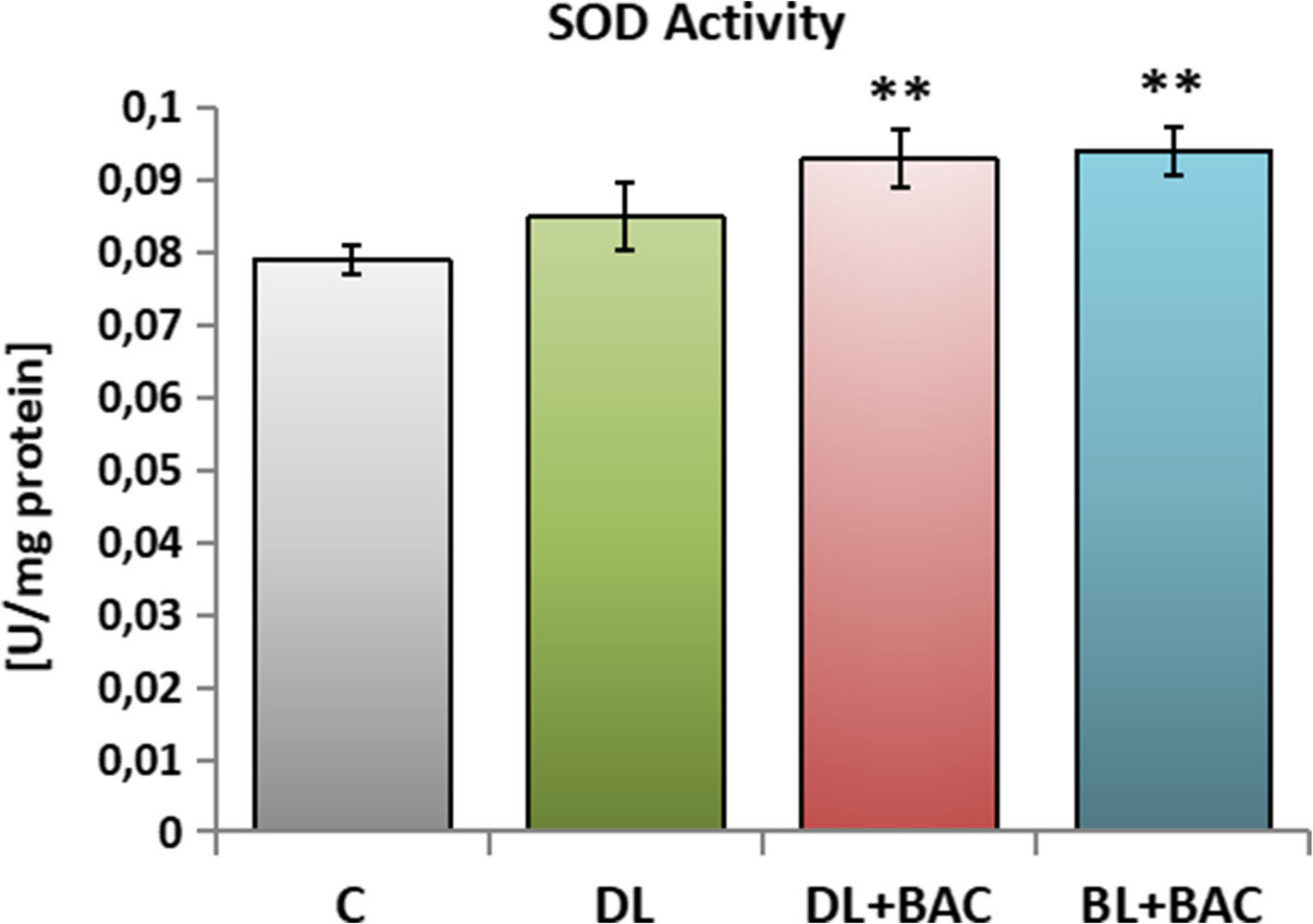
Effect of topical carbonic anhydrase inhibitors (CAIs) on the superoxide dismutase (SOD) activity in the tear film. C – control group (*n* = 25); DL – patients with primary open-angle glaucoma (POAG) using topical dorzolamide (Nodofree, Polfa Warszawa) (*n* = 14); DL + BAC – patients with POAG using topical dorzolamide + benzalkonium chloride (BAC) (Trusopt, Santen) (*n* = 16); DL + BAC – patients with POAG using topical brinzolamide + BAC (Azopt, Novartis) (*n* = 17). Results are presented as means ± SEM. ** *p* < 0.01 – statistically significantly different from the C group

The superoxide dismutase (SOD) activity was slightly higher in group DL when compared to the group C, but these differences were not statistically significant. This parameter was significantly higher in the group of patients using topical BAC-preserved eye drops (group DL + BAC and BL + BAC) when confronted with the control group. Moreover, in these groups the activities of SOD were higher than in the tear film of patients using the topical preservative-free dorzolamide, yet statistically insignificant (Fig. 2).

**Fig. 3:**
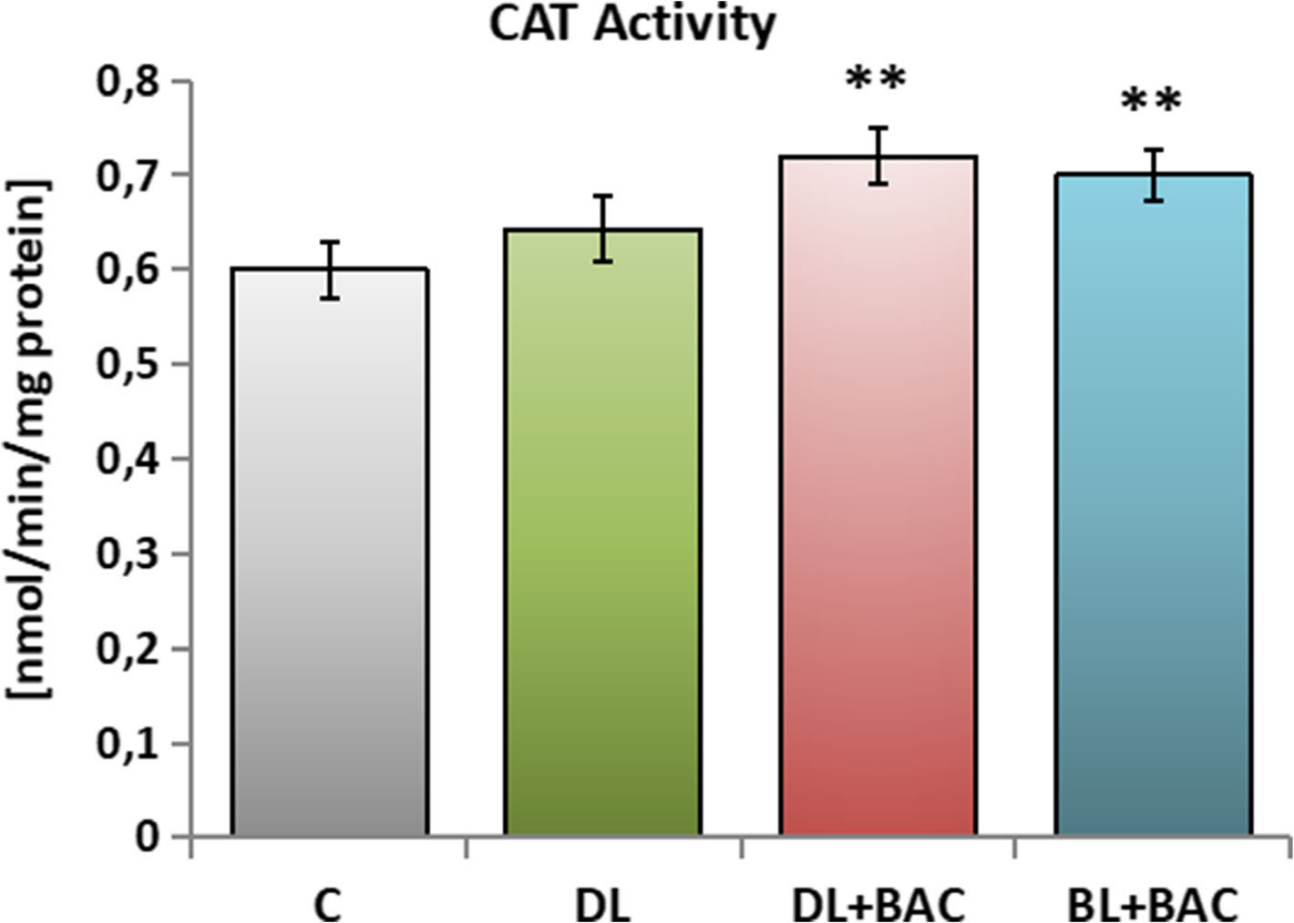
Effect of topical carbonic anhydrase inhibitors (CAIs) on the catalase (CAT) activity in the tear film. C – control group (*n* = 25); DL – patients with primary open-angle glaucoma (POAG) using topical dorzolamide (Nodofree, Polfa Warszawa) (*n* = 14); DL + BAC – patients with POAG using topical dorzolamide + benzalkonium chloride (BAC) (Trusopt, Santen) (*n* = 16); DL + BAC – patients with POAG using topical brinzolamide + BAC (Azopt, Novartis) (*n* = 17). Results are presented as means ± SEM. ** *p* < 0.01 – statistically significantly different from the C group

The catalase (CAT) activity, similarly to SOD, was significantly higher in the groups DL + BAC and BL + BAC in comparison with group C (Fig. 3).

**Fig. 4:**
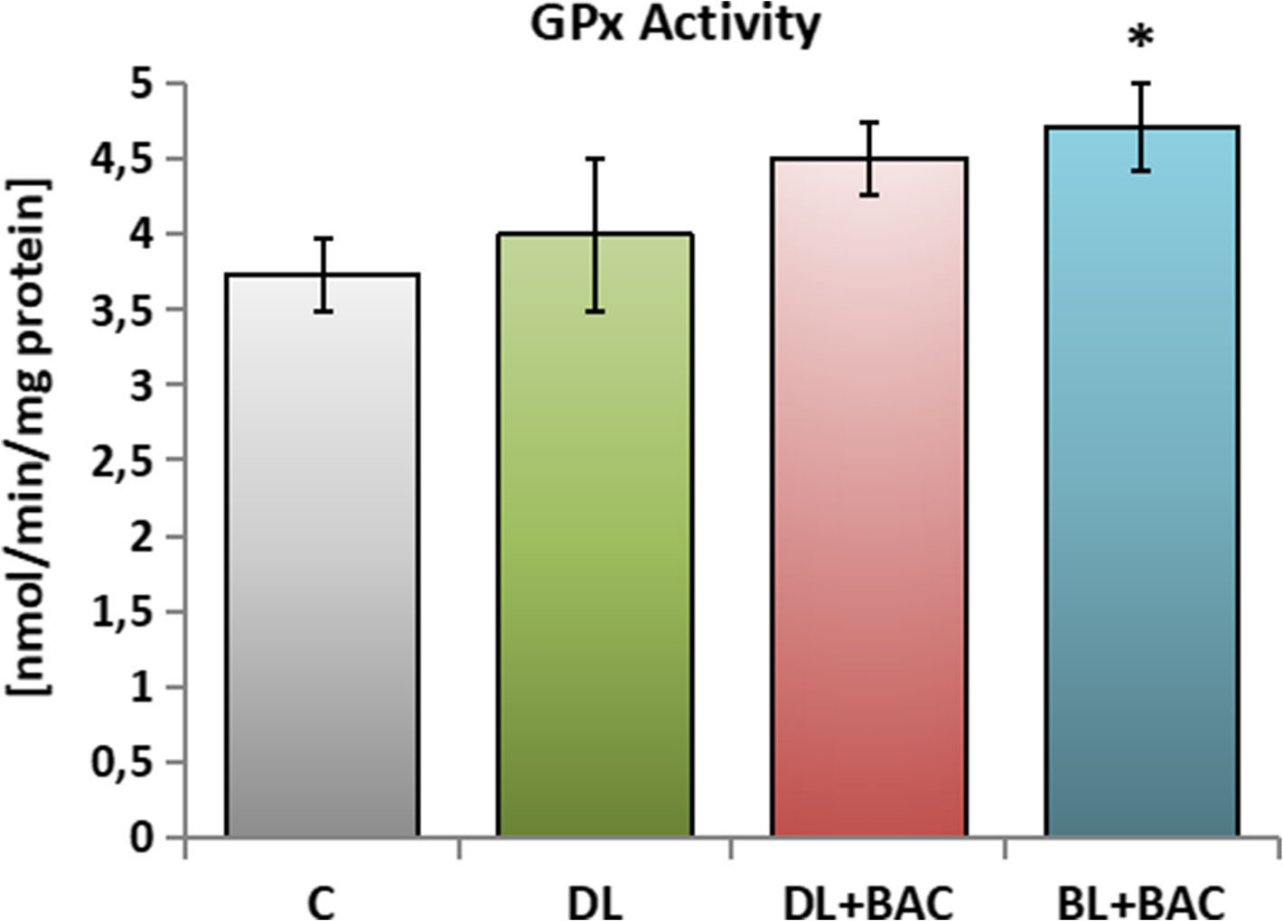
Effect of topical carbonic anhydrase inhibitors (CAIs) on the glutathione peroxidase (GPx) activity in the tear film. C – control group (*n* = 25); DL – patients with primary open-angle glaucoma (POAG) using topical dorzolamide (Nodofree, Polfa Warszawa) (*n* = 14); DL + BAC – patients with POAG using topical dorzolamide + benzalkonium chloride (BAC) (Trusopt, Santen) (*n* = 16); DL + BAC – patients with POAG using topical brinzolamide + BAC (Azopt, Novartis) (*n* = 17). Results are presented as means ± SEM. * *p* < 0.05 - statistically significantly different from the C group

Regarding glutathione peroxidase (GPx), this parameter was not statistically significantly higher in the group DL + BAC in comparison with the group C. However, the activities of this enzyme were significantly higher in the group of patients using BAC-preserved brinzolamide (group BL + BAC) (Fig. 4).

### Effect of the topical CAIs on the nonenzymatic oxidative stress parameters content in the tear film

The advanced oxidation protein products (AOPP) was found to be statistically significantly higher in the groups DL + BAC and BL + BAC when confronted with group C. As compared to the result obtained in the group DL, the AOPP content was significantly higher in the group DL + BAC and not significantly higher in the group BL + BAC (Table 1).

**Table 1:** Effect of topical dorzolamide, benzalkonium-preserved dorzolamide, and benzalkonium-preserved brinzolamide on the advanced oxidation protein products (AOPP) and total sulfhydryl (-SH) groups content in the tear film

Parameter/group	C	DL	DL+BAC	BL+BAC
**AOPP** [nmol/mg protein]	3.64 ± 0.21	3.70 ± 0.23	4.43 ± 0.21*	4.33 ± 0.18*
**Total -SH groups** [μmol/ml]	0.731 ± 0.018	0.698 ± 0.016	0.663 ± 0.014*	0.664 ± 0.012*

C - control group (*n* = 25); DL - patients with glaucoma using topical dorzolamide (*n* = 14); DL+BAC - patients with glaucoma using topical dorzolamide + benzalkonium chloride (BAC) (*n* = 16); BL+BAC - patients with glaucoma using topical brinzolamide + BAC (*n* = 17)

Results are presented as means ± SEM. * *p* < 0.05 - statistically significantly different from the C group

Regarding the total sulfhydryl groups content, in both group of patients treated with BAC-preserved medications (groups DL + BAC and BL + BAC), the results were statistically lower than in the group C (Table 1).

### Effect of topical CAIs on the Total Oxidant Status (TOS), Total Antioxidant Resposne (TAR) and Oxidative Stress Index (OSI) in the tear film

The TOS was found to be slightly higher in the group DL in comparison with the group C, but statistically insignificant. However, the TOS values obtained in the groups of BAC-preserved dorzolamide (DL + BAC) and BAC-preserved brinzolamide (BL + BAC) were significantly higher than in the group C (Table 2).

**Table 2:** Effect of topical dorzolamide, benzalkonium-preserved dorzolamide, and benzalkonium-preserved brinzolamide on the Total Oxidant Status (TOS), Total Antioxidant Reactivity (TAR) and Oxidative Stress Index (OSI) in the tear film

Parameter/group	C	DL	DL+BAC	BL+BAC
**TOS** [nmol H_2_O_2_/ml]	0.069 ± 0.003	0.074 ± 0.004	0.083 ± 0.002*	0.085 ± 0.004**
**TAR** [nmol trolox/ml]	0.066 ± 0.003	0.075 ± 0.005	0.068 ± 0.002	0.070 ± 0.002
**OSI**	1.05 ± 0.02	1.03 ± 0.07	1.23 ± 0.05**^^	1.22 ± 0.05**^^

C - control group (*n* = 25); DL - patients with glaucoma using topical dorzolamide (*n* = 14); DL+BAC - patients with glaucoma using topical dorzolamide + benzalkonium chloride (BAC) (*n* = 16); BL+BAC - patients with glaucoma using topical brinzolamide + BAC (*n* = 17)

Results are presented as means ± SEM. * *p* < 0.05, ** *p* < 0.01—statistically significantly different from the C group. ^^ *p* < 0.01—statistically significantly different from the DL group

In the control group, the TAR was recorded to be 0.006 ± 0.003 µmol trolox/ml, while in the groups DL and BL + BAC was slightly higher, but these differences were not statistically significant (Table 2).

The OSI values in the groups DL + BAC and BR + BAC were significantly higher in comparison with group C. Moreover, in comparison with the group DL, this index was statistically significantly higher in group DL + BAC and BL + BAC (Table 2).

There were no significant differences in any of the investigated oxidative stress parameters between females and males.

## Discussion

To maintain the sterility and prolong the duration of bottle use, most eye drops contain preservatives. Among them, the most commonly used is BAC, a quaternary ammonium salt that through its surface-active structure alters the permeability of cell walls and membrane of microorganisms making them unstable and resulting in cell lysis. However, the tensioactive properties of BAC also induce interaction with the lipid layer of the tear film, consequently leading to excessive tear evaporation. Additionally, it has a well-established effect on diminution in the number of goblet cells producing mucin, meibomian gland dysfunction and tear hyperosmolarity. Importantly, the loss of the protective properties of the tear film and the disturbance of homeostasis lead not only to the formation or aggravation of the dry eye syndrome, but also to corneal damage or the spread of cytotoxic inflammatory mediators [4, 7–12].

BAC is typically used in topical ophthalmic preparations in concentrations varying from 0.004 to 0.02 %. However, it has been proven that already at a concentration equal to 0.005 %, BAC shows a toxic effect on ocular structures [4]. It induces morphological and functional changes such as disruption of tight junctions, inhibition of cell migration, cell growth arrest, cytoplasmic damage and apoptosis in a time- and concentration-dependent manner [13–15]. What is more, BAC also shows neurotoxic activity and significantly reduces the density of sub-basal corneal nerves [16].

In addition, BAC increases the expression of chemokines and cytokines receptors, elicits the release of inflammatory cytokines that inhibit the production of neurotransmitters and activity of sensory nerves thus weakens the secretion of the lacrimal gland. Moreover, the tear hyperosmolarity, enhanced by BAC, also intensifies the secretion of inflammatory mediators and potentiates the inflammatory cascade [29]. There is a strong positive correlation between the level of cytokines in the tear film and the OSDI score [30]. It is worth noting, that also among asymptomatic patients an increase in inflammatory biomarkers in ocular surface cells can be observed [31].

The results of clinical studies in which patients with glaucoma were switched from BAC-preserved to alternative preservatives or preservative-free formulations show alleviation of ocular symptoms and signs and improvement in the quality of life. Analysis of two clinical trials conducted among glaucoma patients reporting OSD symptoms showed that 12 weeks after switching treatment from BAC-preserved latanoprost to preservative-free tafluprost, undesirable symptoms had diminished to one-third [32]. It was also shown that the use of Polyquaternium 1 (Polyquad) preserved Travoprost was associated with a significant reduction of adverse effects as compared with the use of BAC-preserved Travoprost. Moreover, in the polyquad group, the OSDI scores were comparable to the control group which received no medications [33]. Among patients using preservatives beta-blockers, after switching to preservative-free timolol, a significant improvement in the tear break-up time and Schirmer’s test results was noted, with a reduction in deleterious effects, which was translated into an improvement in the quality of life [34]. Comparing the effects of preservative-free and BAC-preserved formulations of the dorzolamide/timolol fixed combination (CosoptTM), in the first group the percentage of reported side effects, such as stinging, burning, redness, was lower (16 % vs. 21.5 %, respectively). Also, the punctate corneal epithelial erosions occurred in fewer patients (16.8 % vs. 23.8 %), whereas the efficacy in lowering IOP was equal in both groups [35].

Side effects of BAC as well as CAIs on the various tissues of the ocular surface have been widely described. However, the impact of preserved and unpreserved CAIs on oxidative stress in the tear film still remains an area requiring investigation.

Oxidative stress is a result of an imbalance between reactive oxygen species (ROS) production and antioxidant defense, and its severity may cause, inter alia, ocular surface epithelial damage, premature senescence, inflammation, dry eye syndrome as well as a decrease in aqueous secretory function [36–39]. The tear film, as the first-line barrier of the eye, is the most exposed to the toxic impact of the eye drops and plays a key role in the antioxidant defense grid thanks to its non-enzymatic (L-cysteine, L-tyrosine, glutathione (GSH), ascorbic acid, uric acid, lactoferrin, S100A proteins) and enzymatic (SOD, CAT, GPx) antioxidants [36, 37, 40–42]. The in vitro studies carried out so far have shown that BAC increases the formation of ROS in the conjunctival and corneal epithelium as well as trabecular meshwork cells [43–45]. The free radicals are extremely reactive and can attack all biomolecules, but lipids, forming part of cell membranes, remain the most susceptible to undergo oxidation [37].

In our study, to assess the oxidative stress in the tear film, we used the OSI, which is the ratio of oxidants (represented by TOS) to antioxidants (presented by TAR). In the group of patients using BAC-preserved CAIs, OSI and TOS in the tear film were significantly higher in comparison to the tears of control, non-treated individuals. Moreover, it should be noted that OSI values were higher in the group of patients treated with BAC-preserved CAI than in the group using preservative-free dorzolamide. This observation is consistent with our previous studies, in which we assessed the influence of selected BAC-preserved prostaglandin F2a analogs (PGAs) (latanoprost, bimatoprost), BAC-preserved beta-blocker (BB) (timolol), BAC-preserved alpha 2-agonist (brimonidine) and their unpreserved counterparts [46, 47]. However, for PGAs, OSI and TOS were elevated in all patient groups treated with PGAs, both BAC-preserved as well as preservative-free formulations [46]. The presented results prove that BAC contained in the analyzed formulations contributes to the increase of oxidants in the tear film.

We noticed the reduction in the content of the total -SH groups in the tear film of patients using BAC-preserved CAIs, as compared to the tear film of the control group. After the application of BAC-preserved eye drops, the AOPP content in the tear film was significantly higher in comparison to the results obtained among untreated individuals. The thiol groups compose, among others, glutathione or L-cysteine and by the formation of the reversible disulfide bonds with proteins, protect them from oxidation [41, 48, 49]. The value of AOPP informs about the damage of proteins formed as a result of interaction with ROS [25]. The thiol groups depleted content in the tear film of patients treated with BAC-preserved medications implies the appearance of the oxidative stress in the tear film with the following involvement of nonenzymatic antioxidants in defense. This may also be confirmed by the increase in the AOPP content in the tear film after the application of BAC-preserved CAIs. However, the increase in this indicator was not as high as one might assume. This is likely due to the protective effect of thiol-containing compounds.

In the tear film of the patients using topical medications containing BAC, the activity of SOD and CAT was significantly higher when confronted with the control group. Additionally, the activity of GPx was found to be higher in the tear film of individuals using BAC-preserved brinzolamide than in the group treated with preservative-free dorzolamide. SOD, CAT and GPx are enzymes that play a fundamental role in the first line antioxidants defense. SOD is the most powerful antioxidant in the cell that catalyzes the dismutation of superoxide anion (O_2_) to hydrogen peroxide (H_2_O_2_) and oxygen. CAT and GPx, in turn, transform harmful H_2_O_2_ into water and oxygen, thus complementing the detoxification process started by SOD [37, 42]. Based on the obtained results, as well as the observations made in our previous studies [46, 47], it is reasonable to assume that BAC contained in the eye drops contributes to the formation of oxidative stress in the tear film, and also intensifies the antioxidative defense of the ocular surface.

Nevertheless, our study has some limitations. One of them is the fact that it was impossible to verify whether the patients were adhering to the recommended use of antiglaucoma eye drops two times a day every day for a period of 6–12 months, or whether the drop application technique was correct, which could have contributed to the reduction or lack of activity of the drug. Secondly, the trial included only people who declared that they had not used other drops at that time, but the truthfulness of this statement was also not verified. Moreover, the age range of the study participants was wide, from 18 to 70 years of age. Having said that, the mean age of subjects in each group was similar. It should be borne in mind that oxidative damage accumulates and increases with age, whereas the abilities of antioxidant defenses decrease in parallel, which presumably also applies to the tear film [37, 38]. Additionally, the possible influence of other factors (ultraviolet light, pollutants, chemical compounds, microbial antigens) on the oxidative stress in tears and on the ocular surface cannot be excluded [36]. Finally, other excipients that were found in topical medications, including co-solubilizers, may not have been neutral to redox homeostasis. However, their possible effect was probably minimal [8].

## Conclusions

The above study shows increased oxidative stress in the tear film of patients using BAC-preserved dorzolamide and brinzolamide, which is supported by the increased values of TOS, OSI, AOPP content and the reduced total -SH groups content. Moreover, the increased activity of SOD, CAT and GPx in the tear film of the above group may indicate that BAC, through overproduction of superoxide anions, enhanced the enzymatic antioxidative system response in the tear film. Such changes were not observed in patients treated with preservative-free CAIs.

## Data Availability

The data that support the findings of this study are available on request from the corresponding author MŚ.

## Abbreviations

AOPP: Advanced oxidation protein products
BAC: Benzalkonium chloride
BB: Beta-blocker
CAI: Carbonic anhydrase inhibitor
CAT: Catalase
FBUT: Fluorescein break-up time
GPx: Glutathione peroxidase
GSH: Glutathione
H_2_O_2_: Hydrogen peroxide
IOP: Intraocular pressure
LIPCOF: Lid-parallel conjunctival folds
OSD: Ocular surface disease
OSDI: Ocular surface disease index
OSI: Oxidative Stress Index
PF: Preservative-free
PGA: Prostaglandin F2a analog
POAG: Primary open-angle glaucoma
ROS: Reactive oxygen species
SH: Sulfhydryl
SOD: Superoxide dismutase
TAR: Total Antioxidant Response
TOS: Total Oxidant Status
TP: Total protein
